# An Efficient Framework for Large Scale Multimedia Content Distribution in P2P Network: I2NC

**DOI:** 10.1155/2015/303505

**Published:** 2015-10-29

**Authors:** M. Anandaraj, P. Ganeshkumar, K. P. Vijayakumar, K. Selvaraj

**Affiliations:** Department of Information Technology, PSNA CET, Tamilnadu 624 622, India

## Abstract

Network coding (NC) makes content distribution more effective and easier in P2P content distribution network and reduces the burden of the original seeder. It generalizes traditional network routing by allowing the intermediate nodes to generate new coded packet by combining the received packets. The randomization introduced by network coding makes all packets equally important and resolves the problem of locating the rarest block. Further, it reduces traffic in the network. In this paper, we analyze the performance of traditional network coding in P2P content distribution network by using a mathematical model and it is proved that traffic reduction has not been fully achieved in P2P network using traditional network coding. It happens due to the redundant transmission of noninnovative information block among the peers in the network. Hence, we propose a new framework, called I2NC (intelligent-peer selection and incremental-network coding), to eliminate the unnecessary flooding of noninnovative coded packets and thereby to improve the performance of network coding in P2P content distribution further. A comparative study and analysis of the proposed system is made through various related implementations and the results show that 10–15% of traffic reduced and improved the average and maximum download time by reducing original seeder's workload.

## 1. Introduction and Related Work

P2P network does not require any central coordination by any central authority or server. It is more fault-tolerant and scalable than traditional centralized client server system. When a new node arrives at the network and requests some service, it provides services to other nodes in the network as well to make use of the services provided by other nodes in the network. It increases total system bandwidth and also reduces the server's load. This causes limitless scalability of the system without any need for additional cost [1]. This type of system is especially useful for distributing large scale multimedia content efficiently to a large group of users. Existing research works in P2P content distribution networks are grouped around three broad questions: first, the fundamental performance limits of the network based on its capacity to exchange information over a given network, second, the possibility to design efficient mechanisms that have desirable properties that meet out the practical requirements, and, finally, the ways to monitor, manage, and control the network. The problem formulations in P2P network are around these questions and information flow over networks may be considered to be an optimization problem: we felt that there is a need to improve the throughput as high as possible for a given bandwidth but to reduce the delay for a specified throughput. Network coding has theoretically proven mechanisms for improving the performance of P2P network in terms of both throughput and average download time. Network coding [2] not only allows intermediate nodes to store and forward the received packets, but also allows the intermediate node to perform the arbitrary coding operation on the received packets. Applying network coding to P2P content distribution makes the data scheduling simple and reduces the burden of original seeder. This method also enhances the application-level throughput [3, 4]. It is argued that the computational overhead incurred by the coding and decoding operation may counteract its benefits [5, 6]. It impedes the adoption and deployment of network coding for content distribution.

Several researchers worked on reducing the complexity of network coding [7], so it can be used in both wired and wireless networks. Chunk based network coding has been introduced to reduce coding complexity and by using local rarest scheduling mechanism, it eases the data scheduling [8]. In their approach, the sender may select a segment in a sequential order for generating coded packet and then transmits a new encoded block in the corresponding segment to other peers. Downloader-Initiated Random Linear Network Coding [9] has been proposed to solve the unlucky combination problem in P2P file sharing system. In their approach, each peer needs to examine neighboring peer's current vector before transmitting the coded packet. Upload complete has been taken as a performance metric to evaluate their system. It is referred to as “the number of rounds the network takes for all the peers to obtain the whole information.” Here, all the peers need to share their buffer map (what are all the packets they have in their buffer) to their neighbors more frequently. Most of the existing methods reported here do not control the transmission of redundant traffic flow in the network code based transmission, which increases the network load and reduces the network throughput. This paper attempts to alleviate the problem stated above and contributions of this paper are twofold.

- We propose a novel network coding mechanism for P2P content distribution, which attempts to reduce the complexity associated with network coding and to maximize the innovative information flow in the network. The proposed system is implemented using the NS2 simulator and is evaluated against traditional network coding. The result proves that the proposed system outperforms the traditional network coding counterpart in both average download time and server's workload.
- To reduce the download time further and avoid the flooding of useless coded packets in the network, we propose the intelligent neighbor selection scheme that selects neighbors intelligently and innovative information checker checks whether the received packets are innovative or not. The results show that intelligent neighbor selection helps to improve the efficiency.

Section 2 of this paper describes the proposed system model. In Section 3, the traditional method of network coding and algorithm is discussed.  Section 4 presents the mathematical performance analysis of a P2P file sharing system with traditional network coding. In Section 5, the description of various modules of the proposed system is given. In Section 6, the main experimental results and analysis are provided and finally Section 7 concludes the discussion.

## 2. System Model

We model a P2P network as a directed graph *G* = (*N*, *L*), where *N* represents end hosts and *L* represents link between end hosts. There is a single server *S* ∈ *N*, which wishes to send its content of size *P* to *N* = |*N*| − 1 receiver nodes. A link *L* = (*p*, *q*) exists in *G* if node *p* is able to send packets to node *q* by making use of either UDP or TCP connection. This kind of P2P topology models exists in the literature [10–14]; we make an assumption that any node in the network is able to establish an overlay connection with any other node. The server *S* will not transmit the information as a commodity flow. Instead, the information is transmitted as a sequence of blocks. During the transmission of these blocks between any two nodes, the block is either completely downloaded or not downloaded at all by the receiver. In theory, information flow in the network is synchronous, edge has known capacities, and centralized knowledge about the topology is used to perform the encoding and decoding operations. But in practice, the information travels asynchronously in the form of packets throughout the network. These packets are subject to random delay and loss. Edge capacity of the peers is often not known and also it varies from time to time. It is very difficult to obtain centralized knowledge to perform the reliable content delivery in the dynamic P2P network.

Now let us assume the original content is divided into *n* blocks {*B*_1_,…, *B*_*n*_} for distribution where each block is equally sized. In these packetized workloads, it is sensible to assume that time is measured in terms of slots [15–18]. The length of the time taken by a node to upload one block is treated as a unit. In this case, the upload and download capacity of any node *p* ∈ *N* can be measured in terms of the number of blocks that node can upload and download in each time slot. Each node in the network needs to make decisions on how receivers can be chosen in each slot, which blocks need to be transmitted and how rates are allotted to each receiver for each transmission. This transmission strategy could be either centralized or decentralized. For decentralized strategies, selection of receiver and rate allocation can be either dependent or independent of selection of block [19]. Block selection algorithms can be classified as block scheduling and network coding. With block scheduling, during the transmission of each block, the sender has to select a block from all the original blocks it has received so far and has to transmit it to the receiver. In network coding strategy, the transmission of a block from the source in time slot *t*, the source *S* sends out a coded block *C*_*n*_(*t*). It is a linear combination of the original blocks; that is,

(1) $$\begin{array}{c} C_{n}\left(\;t\;\right)=\sum R_{i}\left(\;t\;\right)B_{i}, \end{array}$$

where *R*_*i*_(*t*) is a random coefficient chosen from the Galois field GF(2^*q*^) for each time slot and each *i* ∈ {1,…, *n*}.

**Figure 1:**
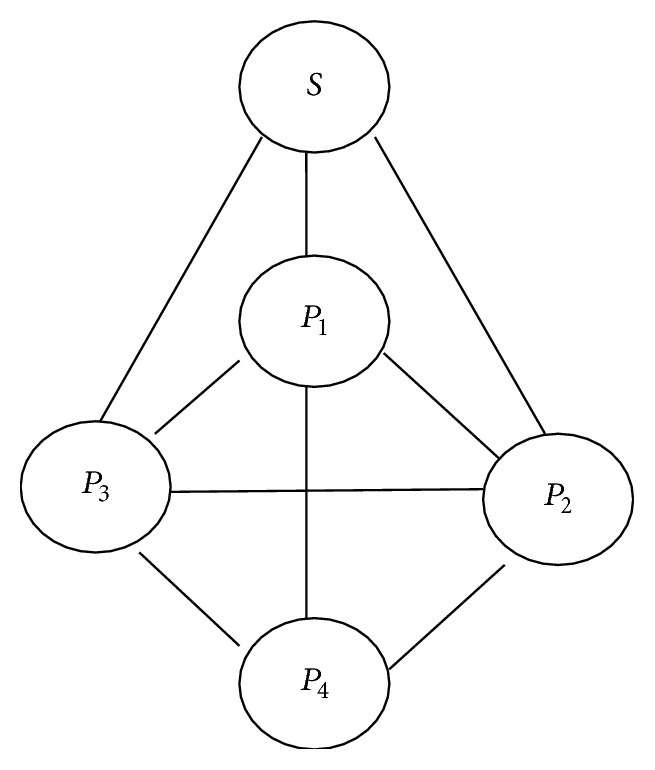
The source *S* distributes packets to the peers *P*_1_, *P*_2_, *P*_3_, and *P*_4_ over overlay network.

For each block transmission of a node *p* ∈ *N*∖{*S*} in time slot *t*, node *p* sends out a coded block *C*(*t*) that is a linear combination of the blocks it has received so far from other nodes. The overhead of transmitting the coding vector along with each coded packet is trivial. If there is 100 extra bits added as a coding vector in the coded packet where the field size is 18, then overhead is 100/1400 = 6%. If we use the network coding for content distribution, the receivers are able to decode the original packets even if the network topology and encoding functions are unknown and nodes and edges are added and removed without giving any notification, link failures, and packet losses with unknown locations. An effective transmission strategy should be decentralized and not required to exchange the state information among the nodes in the network and may remain insensitive to node joins and departures. The proposed system is decentralized one. It is represented in Figure 1, where four peers download the content from one server. After downloading a portion of content from server, peers function in a decentralized manner and collaborate among themselves to download the entire content.

We assume there is a server that maintains the information about of all active peers in a download session. It is similar to the tracker concept in BitTorrent. If any peer wishes to join the session, it contacts the server to get the information about the subset of other peers that are already in the system. Then new peer tries to attach to each of them to create its neighborhood. The impact of peer's joining is less problematic aspect of churn, since it leads to temporary failures like routing inconsistencies or resources which might be temporarily located at a wrong position in the overlay network. The process of peer's leaving the network, however, can result in irreparable damage like loss of the overlay structure or loss of data stored in the overlay. In general, node departures can be classified into friendly leaves and node failures. Friendly leaves make a peer to notify its overlay neighbors to restructure the topology accordingly. Node failures, on the other hand, seriously damage the structure of the overlay by causing stale neighbor pointers and data loss. Since we are primarily focusing on the potential performance gain when reducing the coding density of the network coded packets, we therefore implement the friendly leave model in this paper.

## 3. Traditional Network Coding Mechanism

Each node stores the incoming packets in its buffer. This buffering scheme allows the packets to arrive at the node in asynchronous manner and depart with arbitrarily varying rate, delay, and loss [20]. After each node receiving more than one original or linearly coded packet from other nodes, it selects the linear coefficients in a finite field GF(2^*s*^) in a random manner and combines the packet. The combined packet will be forwarded to other nodes in the network. Encoding vector will be sent within the same packet. It is used to perform the decoding operation at the receiver. All packets related to the same source vectors *V*_1_, *V*_2_,…, *V*_*n*_ are said to be in the same generations. Here *n* represents the size of the generation. All packets belonging to the same generation are tagged with the same generation number. One byte is enough to represent this information in each packet.

**Figure 2:**
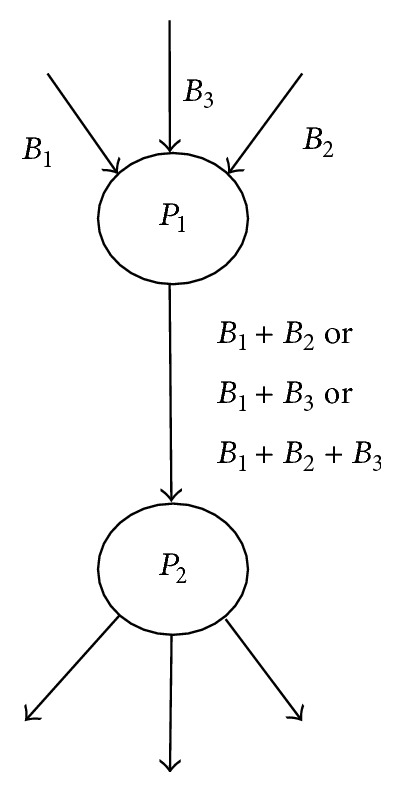
If node *P*_1_ performs coding operation, *P*_1_ can send any linear combination of *B*_1_, *B*_2_, and *B*_3_ to peer *P*_2_.

In Figure 2, peer *P*_1_ has three blocks in its buffer such as *B*_1_, *B*_2_, and *B*_3_. It has different choices to do the coding operation. They are *B*_1_ + *B*_2_, *B*_1_ + *B*_3_, *B*_1_ + *B*_2_ + *B*_3_, and *B*_2_ + *B*_3_.

The following steps are performed in the traditional network coding algorithm.

(1) Each peer needs to store the incoming packet into its buffer until it receives either more than one packet or elapses of prefixed time. The former is done in the synchronous network coding and the latter is done in the asynchronous network coding.
(2) After receiving more than one packet or after elapse of prefixed time interval, each peer combines received packets using random linear combination and sends them out.
(3) After receiving a packet, each peer decides whether to store the packet or to drop it. There are two possible packets each peer receives, one is innovative packet, and another one is noninnovative packet. If the packet is useful or innovative one, then the coding vector of that packet is stored in the matrix. The packet is said to be innovative if it is used to increase the current rank of its own matrix. The packet is said to be noninnovative if it is not used to increase the rank of its matrix. It means that the packet has redundant information and it is not needed to decode the original packets and these packets are dropped. The matrix needs to be updated for the reception of each random linear combination of source packets.
(4) Each peer has to check whether the matrix has full rank (*n*) or a submatrix with full rank (*s* < *n*) that exists or does not for receiving each of coded packets.
(5) If the matrix or the submatrix of peer *S* has full rank, then by using the Gaussian elimination method, peer *S* can decode all or partial number of original packets.

*Definitions*

**Definition 1 .**

A transmitted packet is said to be readily decodable for receiver *R*_*j*_ if the linear combination of the coded packets contains at most one original packet that the receiver has not yet decoded.

**Definition 2 .**

A transmitted packet is called as innovative packet for receiver *R*_*j*_ if it did not contain the packets previously received by *R*_*j*_ in the span of linear combination.

**Definition 3 .**

The receiver *R*_*j*_ realizes a delay of one time slot if it successfully receives a packet that is either noninnovative or not readily decodable.

## 4. Mathematical Analysis of Traditional Network Coding

We assume that packets arrive at each peer in P2P network according to a Poisson process [21] and there are a total of *p* number of peers in the network. The packet arrival rate at each peer is *λ*/*p* so that the total arrival rate of packets is fixed and it is *λ* packets/slot. Every time new packets arrive at the node, it tries to find the opportunity to code the packets together and transmit the coded packet to the next time slot. After receiving the packet in each time slot, one of two possible events may occur: either there is an opportunity to create innovative information flow by combining more than one packet together or it is not possible to create innovative information flow. All the nodes involved in the transmission of useless packets to other nodes are said to be backlogged until it is possible for them to create an innovative packet. Every time a node is involved in transmitting the useless packets to other nodes, it needs to wait for receiving enough packets in its buffer to create innovative information flow. That node has to find a good random combination for generating new coded packets by using previously received packets. This random combination follows a numerical distribution. In traditional network coded P2P transmission, each backlogged peer chooses to transmit the innovative packet with probability *p* and hence the transmission of noninnovative packet with probability (1 − *p*). This network can be described by a Markov chain; *B*_*n*_ is the number of backlogged peers at the end of *n*th slot. To start with, we evaluate the transition probabilities of the Markov chain, *p*_*j*,*k*_.

We note that

(2) Prob⁡m  backlogged  peers  try  to  send  the  non-innovative  packet ∣ Bn=k=kmpm1−pk−m

(3) Prob⁡m  new  arrivals  of  packets ∣ Bn=k=λmm!e−λ.

Using (2) and (3), the transition probabilities can be determined as follows:

(4) pj,k=0for  k<j−1ip1−pj−1e−λfor  k=j−1λk−1k−j!e−λfor  k=j+11−1−pjλe−λfor  k>j+11+λ1−pj−ip1−pj−1e−λfor  k=j.

We define the flow of the steady state behavior of this Markov chain in state *j* as

(5) $$\begin{array}{c} d_{j}=E\left[\;X_{l+1}\;|\;X_{l}=j\;\right]-j. \end{array}$$

If the flow is positive, then the number of backlogged peers will tend to increase, whereas if the flow is negative, the number of backlogged peers will tend to decrease. A flow of zero represents some sort of equilibrium for the Markov chain. Given the proceeding transition probabilities *p*_*j*,*k*_, the flow works out to be

(6) $$\begin{array}{c} d_{j}=\lambda {\left(\;1-p\;\right)}^{j-1}e^{-\lambda }\left[\;ip+\lambda \left(\;1-p\;\right)\;\right]\lambda . \end{array}$$

Assuming that *p* ≪ 1, then we can make use of the approximation (1 − *p*) ≈ 1, and (1 − *p*)^*j*^ ≈ *e* ^−*ip*^. The following is the simplified expression for the flow:

(7) $$\begin{array}{c} d_{j}=\lambda -f\left(\;j\;\right)e^{-f\left(j\right)}\;\text{where }f\left(j\right)=\lambda +ip. \end{array}$$

**Figure 3:**
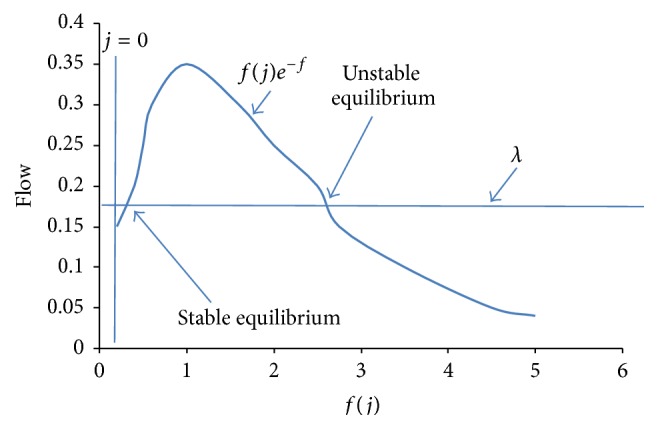
Flow Vs *f*(*j*).

The parameter *f*(*j*) is interpreted as the average number of the innovative packet transmissions per slot given that there are *j* backlogged states. To understand the significance of this result, the two above stated terms in the expression for the flow are plotted in the graph. The first term in the above flow equation (*λ*) has the interpretation of the average number of new packet arrivals. The second term, *f*(*j*)*e* ^−*f*(*j*)^, is the average number of possibilities of creating innovative packets. For smaller value of *j*, the rate at which packet arrives is greater than the rate at which packet departs and also the number of nodes in backlogged states tends to increase. For modest values of *j*, the departure rate is greater than the arrival rate and the number of backlogged states tends to decrease. The flow of the Markov chain tends to be stable around the point marked stable equilibrium in the graph. For large *j*, the flow tends to be positive again. If the number of backlogged states ever becomes large enough to make the system to the right of the point marked unstable equilibrium in the graph shown in Figure 3, then the number of backlogged nodes will tend to increase without bound and the network will become unstable. The value of the *λ* represents the throughput of the system. If we make the value of *λ* that is greater than the peak value of *f*(*j*)*e* ^−*f*(*j*)^, then the flow will always be positive value and the network will be unstable from the start. This maximum throughput occurs when *f*(*j*) = 1 and has a value of *λ*_max_; we can get the system to operate near the state equilibrium. The system will flow into the unstable region sooner or later. The lower the arrival rate is, the longer the system will take to reach the unstable region. Traditional method of network coding is an unstable process if it is not implemented carefully. Various modifications are needed in the traditional method of network coding to exhibit stable behavior.

## 5. Proposed System Architecture

**Figure 4:**
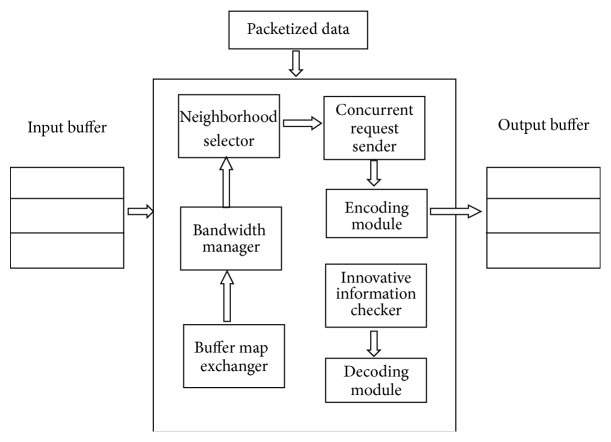
Proposed system architecture.

Most of the optimal decentralized algorithms based on block scheduling require an exchanging of buffer map among them very often. This buffer map contains information about the set of blocks the peers have in their buffers at present. The size of the buffer map increases rapidly as the size of the content increases. The frequent and accurate buffer state exchanges are always difficult to perform in mesh-based P2P network [3]. This problem can be eliminated with the aid of network coding and by making the protocol more robust against peer churn.  Figure 4 shows the architectural overview of the proposed system. This proposed system consists of five different modules such as bandwidth manager, buffer map exchanger, neighboring peer selector, encoding module, data request sender, innovative information checker, and a decoding module. The architectural overview of the proposed system is presented in Figure 4. Description of the various modules of I2NC is given in the next section.

### 5.1. Bandwidth Manager

This module is used to manage the uplink and downlink capacity of each peer in the network. Since a server is not able to serve a large scale content to the multiple users simultaneously, it divides the file into *n* original blocks *F* = (*B*_1_, *B*_2_,…, *B*_*n*_) and uploads the coded blocks to different clients in the P2P network. The peers in the network need to exchange those coded blocks or newly generated coded blocks among themselves to obtain the entire content. Here the server's load is reduced and this makes the client also involved in the process of downloading. In the proposed system, each peer knows their small subset of the nearby peers, which have the common interest to download the large scale content. This relationship is said to be symmetric; that is, if peer *P*_1_ is the neighborhood of *P*_2_, then *P*_2_ is also a neighborhood of *P*_1_. Each peer exchanges the metadata only with its nearby or neighboring peer only. The number of neighbors is normally a small value. Peers can download/upload the files with their neighborhood peers concurrently. The number of blocks that can be downloaded/uploaded by the peer is propositional to its download/upload capacity. In the broadband Internet connections, bandwidth bottlenecks occur at the edge of the network rather than in the core of the network. The upload and download rates of each node are constrained by its upload capacity and download capacity UL_*p*_, DL_*p*_ respectively. We assume download capacity is greater than the upload capacity of all the nodes in the network. It is also assumed that each node in the network has its own upload and download capacity constraints. The content distribution ability of P2P network can be evaluated by the achievable download rates and download finish times. If the large scale content is to be distributed, then we define the download rate of each peer in the network as

(8) $$\begin{array}{c} {\text{DR}}_{i}=\underset{n\to \infty }{lim}\frac{n}{T_{p}\left(n\right)},\;p=1,2,\ldots ,N. \end{array}$$

Here *T*_*p*_(*n*) is the time the node *p* takes to complete the downloading of entire content. The content contains *n* number of blocks. Each node in the network is constrained to its download capacity and the maximum rate at which the sender can send out the blocks. The sum of download rates of all the peers in the network cannot exceed the total upload capacity of the network. The achievable rate must satisfy the following condition:

(9) DRp≤Min⁡ULi,DLj,j=1,2,…N,∑i=1pDRi≤∑i=1NULi+UPi.

In the above equation UP_*s*_ stands for the maximum rate at which source can send out the content. We consider a homogeneous network where upload capacity is equal to the totality of upload capacity of all peers in the network. Our proposed system can be applied to any finite download capacity values where download capacity is always greater than or equal to the upload capacity of all peers in the network.

### 5.2. Buffer Map Exchanger

The following steps should be carried out in order to exchange the buffer map among the peers. The major objective of this module is to reduce the amount of control information flow in the network.

1. If any peer wants to be connected within the network, it approaches to the tracker like BitTorrent system and gets the list of peers currently downloading the content.
2. The small set of neighboring peers should be selected by the newly connected peer. The neighboring peer selection module performs this task. The number of neighboring peers, to which it is connected, depends on its upload and download capacity.
3. Each peer incrementally changes its neighboring peers by calling its neighboring peer selector.
4. Each peer needs to broadcast the global encoding vectors of its newly received innovative blocks to all its neighboring nodes. Each peer does not require the periodical exchange of buffer map information. It is done only when the peer communicates with the new peer.
5. This global encoding vector is used to find the best optimal packet selection to carry out the coding operation.

Each peer has to maintain a matrix that contains coding coefficients of received coded packet from its set of neighbors. This is a global coding vector for all the available coded blocks in that peer at present. It evolves over the time.

### 5.3. Neighboring Peer Selector

This module performs the selection and maintenance of neighboring peers. Each peer belonging to an overlay network may select neighboring with no regard for the underlying physical network properties. It should select such neighbors that are close with respect to some network metric. Some popular metrics are network latency, RTT, and geographic distance. When peer newly joins the network, it will contact tracker to obtain the list of peers currently downloading the content and its IP addresses. The proposed neighbor selection method consists of two stages. In the first stage, we find *n* initial set of neighbors, according to the idea borrowed from [22]. In this stage, we make use of IP addresses to select the nearest neighbors. We use the longest common IP prefix length as measure proximity among neighbors. In the second stage, we find the proper neighbors using the threshold value. It is calculated based on the packets those peers have currently. The neighboring peer selection has the following components along the calculation of nearby nodes in the network. These components are used to calculate the threshold value.

1. Information about neighboring peers of upstream peer is communicated with downstream peer. This describes not only connectivity to various peers, but also available resources such as bandwidth. Instead of describing the network topology completely, less overhead is incurred by proving partial information in the form of coefficient vectors. It is formed by random network coding. This is called announcement vector. These vectors are linearly indicating the availability of link disjoint path to various peers. The linear dependencies are likely to share a bottleneck link.
2. Downstream peer requests information from upstream peer. Each peer initiates set of requests which will be propagated to upstream peer along disjoint paths. This path is found by using announcement vector.

The maximum number of neighbors for each peer should be less or equal to the ratio between capacity of the peer and minimum allocation for either downstream or upstream bandwidth (see Pseudocode 1).

### 5.4. Encoding Module

Avoiding cycles in network code based data transmission is more complicated than preventing cycles in traditional routing since the same data need to be transmitted over multiple paths in different linear combinations in network code based transmission. The following are the important components of the encoding module.

(1) Information requested by one peer may be useful to another. Requests are propagated to upstream peer if it has useful information. Any peer seeks a packet from another peer whose coefficient vector is linearly independent of a set of vector *S* which describes using vector *u* in the null space of *S*. A vector whose dot product with *u* is nonzero is called independent vector in *S* ^2^. It is termed as opposite to *u*.
(2) Intermediate peers send appropriate information in response to request. A peer *P* transmits on each of its outgoing links *O* a random linear combination of a subset of its input information vectors. The subset is selected to satisfy the request received on *O*.

Each peer stores an announcement vector and a current vector for each of its neighboring incident connections. The announcement vector *A*(*c*) of each connection *c* is formed at its neighboring peers as a random linear combination. It is specified by randomly selected coefficients *F*_*i*,*c*_ *A*(*i*) of announcement vectors of its incoming connections

(10) $$\begin{array}{c} R\left(\;c,r\;\right)=\sum_{i:d\left(i\right)}\;=o\left(\;c\;\right)F_{i,c}A^{\prime }\left(\;i\;\right) \end{array}$$

and it is communicated to its neighboring peer. The announcement vector of a connection is updated if and only if the announcement vector of one of its incoming connections changed. The announcement vector will stabilize as long as there is a change in network topology.

*R*(*c*, *r*) is called reduced vector and is set for (connection, receiving peer) pairs (*c*, *r*). The connection *c* is in the set *Q*(*r*) of connection traversed request form peer *r*. Otherwise, for *c* not belonging to *Q*(*c*), *R*(*c*, *r*) is set or updated if the reduced vector *R*(*c*′, *r*) of an incident incoming link *c*′ is set or updated. It is performed according to the following:

(11) $$\begin{array}{c} R\left(\;c,r\;\right)=\sum_{i:d\left(i\right)}\;=o\left(\;c\;\right)F_{i,c}A^{\prime }\left(\;i,r\;\right), \end{array}$$

where *A*′(*i*, *r*) = *A*(*i*, *r*) if *R*(*i*, *r*) has been set; otherwise *A*′(*i*, *r*) = *A*(*i*). The encoder builds a network coding solution by changing the current vectors. The current vector of each connection is set to zero vectors. It will be updated in response to requests and whenever the current vector set by the encoder gives the linear combinations of data to be sent on each connection.

### 5.5. Concurrent Request Sender

Before any peer wants to send a request to download a data block, it first checks its neighbors to create lists which can send innovative blocks for itself at that moment. It can be done by examining the current global coding vector of each peer in the network. This information is already available in that peer. If the peer is newly joined, then the following steps have to be performed.

1. Request the neighboring peer selector to select the set of neighbors for that peer.
2. Its available bandwidth is divided into two sets; one is used for downloading and another one is used for uploading. But initially for short duration all available bandwidth is devoted to download since it does not have any packet to upload.

Content request is made simultaneously to all its neighboring nodes. Different combinations of useful coded packets from different peers are requested simultaneously.

### 5.6. Innovative Information Checker

Innovative information checker module is used to find whether the received coded block is useful to the downloaded peer or not. After receiving the coded block from its neighbors, each peer checks whether it is innovative one by using the following equation:

(12) $$\begin{array}{c} I=\left|\begin{array}{c} B_{j}\left(t\right)\\ B_{k}^{\left(j\right)}\left(t\right) \end{array}\right|-\left|\;B_{j}\left(\;t\;\right)\;\right|, \end{array}$$

where | | is denoted as rank of the matrix. *B*_*j*_(*t*) is denoted as a global coding coefficient of all the available blocks in peer-*j* at time *t*. *B*_*k*_^(*j*)^(*t*) is denoted as a coding coefficient of block received from its neighboring peer-*k* by peer-*j*.

In the above equation, *B*_*j*_(*t*) and *B*_*k*_^(*j*)^(*t*) are maintained in matrix form. If the value of *I* is greater than zero, then downloaded block is an innovative one. The downloaded block should be kept in its buffer. This block will be used when that peer performs the decoding operation. If the value of *I* is less than zero, then peer decides to discard the downloaded block. This block is not at all used while performing the decoding operation.

### 5.7. Decoding Module

Finally, each peer has to perform the decoding process in order to obtain the original source packets. *T*(*c*_1_),…, *T*(*c*_*n*_) are the received coded symbols. It contains global coding coefficients. The coding coefficient describes the coding operation performed in that received coded symbol. Each peer is able to decode the source packets *p*_1_,…, *p*_*n*_, as long as the matrix *G*, formed by the global encoding vector, has full rank *n*. Consider

(13) Tc1⋮Tcn=g1c1⋯g1cm⋮gmc1⋯gm1cnp1⋮pn=gp1⋮pnp1⋮pn=G−1Tc1⋮TCn.

## 6. Experimental Results and Analysis

In this section, let us investigate the performances of different content distribution schemes and compare those with the proposed framework. Nonnetwork coding and random linear network coding are considered to compare with I2NC. Each time simulation starts with one node having the entire content, called a seeder. We created the P2P network, which consists of the maximum of 100 numbers of nodes. We distributed the file of size 1 GB. In addition, the 1 GB file is divided into 512 KB blocks, which are grouped into 4–12 MB fragments. As any pair of peers can link up, the bandwidth of each link is limited to 256 kbps. The number of uplinks and downlinks is also restricted. Each node can have a maximum of five uplinks and downlinks. The innovative information checker module is responsible for detecting the linear dependence of received packets. It will choke an upstream peer for 20 seconds in case it detects continuous linear dependence from the upstream peer. The interarrival times of peer join events are modeled as a Poisson distribution (*λ* = 0.5) and new nodes are not allowed to join the network when the network has already 100 peers. The lifetime of each peer is uniform, which may be the maximum of 50 s or minimum of 100 s. The following table shows the experimental setup parameters.

The following systems are considered to compare the performance of the proposed system.

*Nonnetwork Coding for Content Distribution (NNC)*. The origin server distributes the large scale content to all requesting clients. Further improvement in this scheme is that the origin server may distribute the content to the edge server from where the client can download it instead of downloading from the main server. In this scheme, client measures RTT with selected origin servers to find the nearest edge server so that the communication latency is reduced.

*P2P-Traditional Network Coding Mechanism (TNC)*. The origin server has generated network coded packets and then it sends them to all the participating peers in the network. Thus, for each chunk c, each peer keeps a fraction of random network coded packets. Peers exchange the coded packet among themselves. Upon receiving enough coded packets any peer can reconstruct the original file.

The following metrics are used for evaluating the performance of the proposed system.

1. Packet redundancy: When network coding is not used, packet redundancy is measured by the expected number of times each packet travels in a domain. When network coding is used, this metric is measured by dividing the total amount of network traffic by the minimum amount of network traffic in the ideal case. Ideally, only one copy of the original file has to flow in a network and all the peers can then collect and reconstruct the original file through cooperation.
2. Average packet distribution time: It is measured by the average time all the participant peers take to complete the downloading of enough coded packets to reconstruct the original file.
3. Maximum download time: It is measured by the maximum amount of time for a peer to complete its download.
4. Failure rate: It is number of peers unable to finish their download due to missing of some blocks.
5. Server Load: It is number of packets provided by the server to make all the participant peers in the network finish their downloading. This metric justifies the benefits of P2P network for content distribution instead of traditional client server approach.

Table 1 represents the parameter taken for experimental setup.

### 6.1. Impact of Number of Peers in the Network

**Figure 5:**
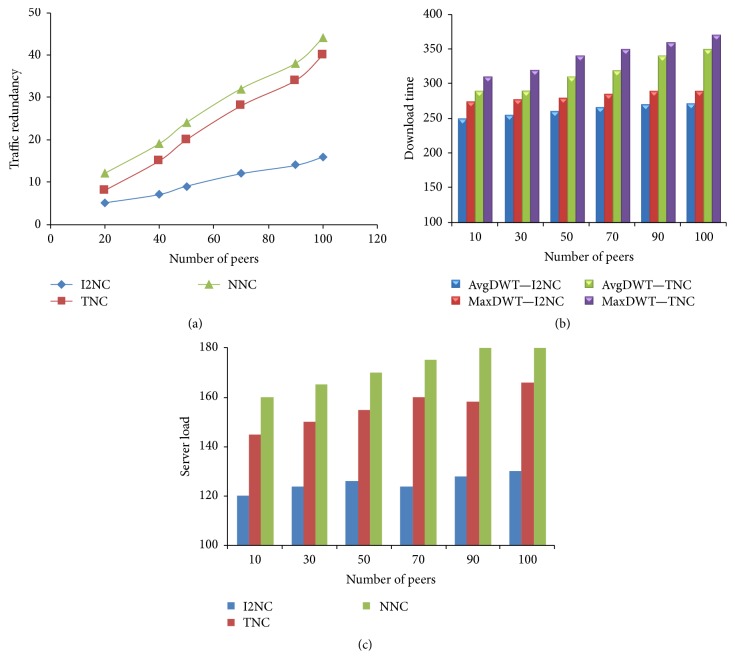
(a) Traffic redundancy. (b) Packet distribution time. (c) Server's workload.

The graphs in Figures 5(a), 5(b), and 5(c) show how the number of peers affect the traffic redundancy, download time, and server's workload. The number of peers in the network is described as a density of the overlay network. We model the density of overlay network by changing the average number of peers in the network. The number of peers is increased from 20 to 120. In Figure 5(a), it is shown that I2NC can effectively reduce traffic redundancy. This is not possible in the traditional system when the number of peers in the network is increased. As the overlay network becomes dense, the traffic redundancy increases in both TNC and NNC schemes. More balanced and nonredundant block distribution can be achieved in I2NC which increases the usability of data blocks for neighboring peers. It further improves the utilization of the links between peers and also reduces server's workload.

### 6.2. Impact on Number of Neighboring Peers

**Figure 6:**
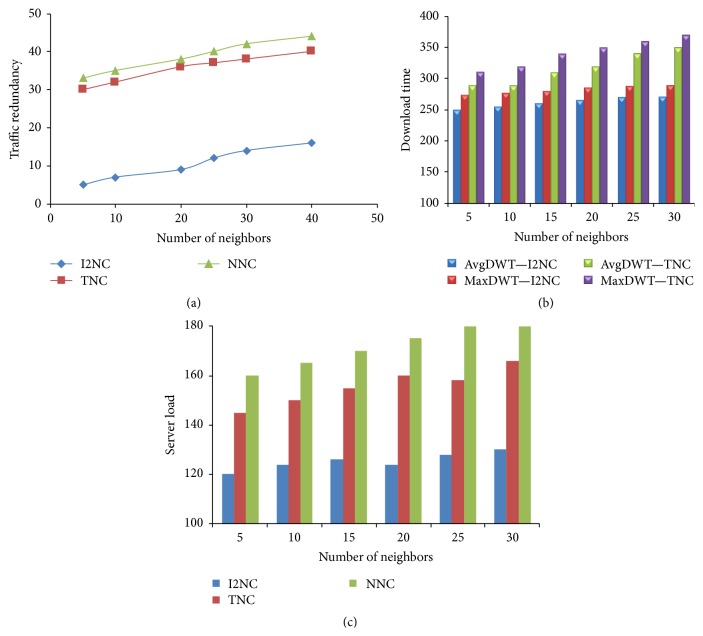
(a) Traffic redundancy. (b) Packet distribution time. (c) Server's workload.

Figures 6(a), 6(b), and 6(c) show how the number of neighbors affects the traffic redundancy, download time, and server's workload. It is shown that the traffic redundancy decreases slightly as the average number of neighbors increases in I2NC. However, in TNC and NNC, a slight increase in traffic is observed as the overlay network becomes dense. With I2NC, when the network gets larger number of peers, the opportunity for a peer to get innovative blocks from its neighbor increases slightly.  Figure 6(b) shows that the average distribution time slightly decreases as the overlay network becomes dense.  Figure 6(c) proves the consistently lower server cost of I2NC than NNC and TNC.

### 6.3. Impact of Coding Density

**Figure 7:**
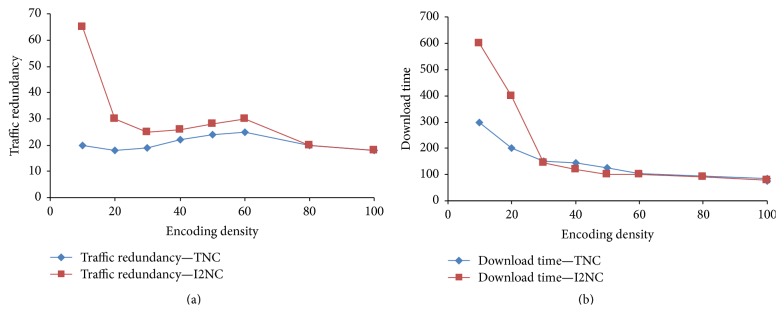
(a) Impact of encoding density in traffic redundancy. (b) Impact of encoding density in download time.

Coding density (*D*) means the number of blocks that are used to produce a coded block. It relates to the end user's coding overhead directly. If the coding density is lesser or the value of *D* is smaller, then reduce the coding overhead. But it has a negative impact on traffic reduction. It reduces the probability to find the innovative packets from its neighboring peers and also each peer has less chance to produce linear independent packet for the requesting peers. If there are more number of packets combined with the coded packet, it may reduce the traffic redundancy. But it increases the coding complexity. The optimal value of *D* needs to be selected. Hence, near optimal performance could be achieved. It is reported in Figures 7(a) and 7(b). It is drawn by taking traffic redundancy in *y*-axis and encoding density in *x*-axis.

The extended download time is largely due to the generation of linearly dependent blocks with lesser coding density. It unnecessarily wastes the network resources. One way to reduce the probability of generating the linearly dependent blocks is to share the buffer map information effectively. In the proposed system, buffer map manger improves the efficiency of exchanging buffer map information.

## 7. Conclusion

Avoiding cyclic transmission of data in network code based data transmission is more complicated than preventing cycles in traditional routing since the same data need to be transmitted over multiple paths in different linear combinations. In this paper, we have examined the effectiveness of network coding mechanism for P2P content distribution with mathematical analysis and proposed an effective framework called intelligent-peer selection and incremental-network coding (I2NC) which avoids cyclic transmission of the same coded packet in network code based content distribution in P2P network. Extensive simulation results given above prove that I2NC can reduce the interdomain P2P traffic by over 15% and reduce 20% of packet distribution time compared to the traditional network coding and nonnetwork code based transmission. Average download time for each peer to download the most needed coded packets is reduced due to the intelligent neighborhood selector and concurrent request sender module. Further, attempts are made to implement the incremental coding mechanism to increase the probability of generating innovative packet. In the proposed system, it is assumed that nodes are all homogeneous and links are error-free and symmetric. In future, security features and heterogeneous network environment will be taken up for consideration.

## Figures and Tables

**Pseudocode 1:**
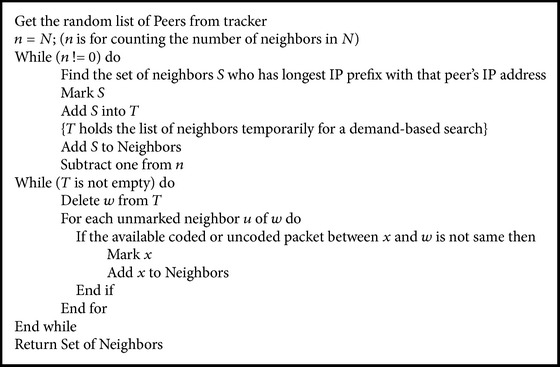
Pseudocode for neighbor selection.

**Table 1:** Experimental parameters.

Configuration parameters	Values
File size	1 GB
Number of blocks in each file	32
Block size	512 KB
Maximum number of peers	100
Peer lifetime	50 or 100 s
Maximum number of uplinks	5
Maximum number of downlinks	5
Bandwidth of each up/downlink	256 kbps
Packet's arrival rate	Poisson distribution (*λ* = 0.5)
